# A system for equitable workload distribution in clinical medical physics

**DOI:** 10.1002/acm2.13460

**Published:** 2021-10-25

**Authors:** Minsun Kim, Eric Ford, Wade Smith, Stephen R. Bowen, Sarah Geneser, Juergen Meyer

**Affiliations:** ^1^ Department of Radiation Oncology University of Washington Medical Center Seattle Washington USA; ^2^ Department of Radiation Oncology Seattle Cancer Care Alliance Seattle Washington USA

**Keywords:** equity, leadership, workload distribution

## Abstract

**Background:**

Clinical medical physics duties include routine tasks, special procedures, and development projects. It can be challenging to distribute the effort equitably across all team members, especially in large clinics or systems where physicists cover multiple sites. The purpose of this work is to study an equitable workload distribution system in radiotherapy physics that addresses the complex and dynamic nature of effort assignment.

**Methods:**

We formed a working group that defined all relevant clinical tasks and estimated the total time spent per task. Estimates used data from the oncology information system, a survey of physicists, and group consensus. We introduced a quantitative workload unit, “equivalent workday” (eWD), as a common unit for effort. The sum of all eWD values adjusted for each physicist's clinical full‐time equivalent yields a “normalized total effort” (nTE) metric for each physicist, that is, the fraction of the total effort assigned to that physicist. We implemented this system in clinical operation. During a trial period of 9 months, we made adjustments to include tasks previously unaccounted for and refined the system. The workload distribution of eight physicists over 12 months was compared before and after implementation of the nTE system.

**Results:**

Prior to implementation, differences in workload of up to 50% existed between individual physicists (nTE range of 10.0%–15.0%). During the trial period, additional categories were added to account for leave and clinical projects that had previously been assigned informally. In the 1‐year period after implementation, the individual workload differences were within 5% (nTE range of 12.3%–12.8%).

**Conclusion:**

We developed a system to equitably distribute workload and demonstrated improvements in the equity of workload. A quantitative approach to workload distribution improves both transparency and accountability. While the system was motivated by the complexities within an academic medical center, it may be generally applicable for other clinics.

## 1 INTRODUCTION

Medical physics is an impactful and rewarding career path to maintain and improve the quality and safety of healthcare services and patient care. In the US healthcare system, clinical providers have a structured system to support reimbursement, which quantifies effort with a metric called relative value unit (RVU). RVUs define the value of a service or procedure relative to all services and procedures. Though there exist issues with this system,^1^, ^2^, ^3^, ^4^ it provides an important benchmark that underlies a substantial portion of the business operations of healthcare at present. However, no such system exists for medical physicists in the therapy or diagnostic realm. This presents many challenges. Without a meaningful metric for clinical effort, discussions of staffing and workload become difficult and it is very challenging to ensure equity in terms of the distribution of clinical tasks.

Only a few approaches to account for the clinical effort and variability in medical physics have been described in the literature,^5^, ^6^, ^7^ and they mainly focus on staffing levels and scheduling rather than workload assignment. On the other hand, several studies in nonclinical academic environments have shown that heavier workloads in tasks such as teaching, administrative service, and mentoring can cause a gender or racial disparity regarding promotion. This might partially explain the scarcity of female or underrepresented minorities, especially at the full professor rank.^8^, ^9^, ^10^, ^11^ Some efforts to achieve a more equal distribution of faculty workload have been made in STEM (Science, Technology, Engineering, & Math) academic departments, where the goal of the intervention was to fairly distribute the service and teaching workload.^9^ This multi‐institutional group conducted a randomized trial and reported positive outcomes from the intervention group in terms of faculty satisfaction and awareness.

In the medical physics context, inequities in clinical workload can have negative career impacts, especially in an academic medical physics setting where research productivity is an important dimension for promotion. An unfair distribution can also negatively affect an individual's success and satisfaction in the workplace. This issue is more pronounced when there is a lack of transparency and accountability for everyone's various responsibilities. To ensure a fair and equitable workload distribution, it is important to have a transparent and all‐encompassing approach, which considers all aspects of clinical service. This is challenging as medical physics comprises not only routine tasks but also numerous complex and diverse special procedures. Furthermore, physics efforts and responsibilities are dynamic and ever‐changing,^12^ and therefore a constant reassessment of tasks and assignments is required. This adds to the complexity that medical physics leaders experience in assigning a fair workload distribution across individuals. One of the roles for medical physics leadership is to ensure a fair workload distribution amongst its members with respect to the coverage of clinical duties.

The purpose of the present study is to develop and examine a system designed to improve equitable distribution of clinical workloads among the medical physics team members in radiation oncology while also increasing transparency and accountability. Using a quantitative metric, this system is designed to incorporate various types of tasks and changes in practice patterns. We present the comparison of the workload distribution for one medical physics group before and after implementation of the system.

## 2 METHODS AND MATERIALS

### 2.1 Effort assessment

At the direction of departmental leadership, a committee was created consisting of four physicists whose purpose was to assess and quantify the current workload distribution within the department. We first collected a list of clinical tasks assigned to eight physicists deployed across two clinical sites of practice. The tasks included physics plan and chart review; procedures such as intravascular brachytherapy or prostate seed implants; daily, monthly, and annual quality assurance (QA); and other tasks. We also gathered information on each physicist's clinical full‐time equivalent (cFTE) deployment, which is the percentage of every physicist's time devoted to the clinic and does not include administrative components or research time allocated from grant funding. We then conducted a survey of 11 physicists to estimate the amount of time typically spent on each of the tasks. As part of the survey, we defined the exact scope of each task in order to accurately quantify the time requirements. This was a key step because a different interpretation or understanding of the task can lead to variability in time estimates from different physicists. Survey data were collected from a range (2–11) of people who were familiar with each task in order to reduce bias in the estimates.

The total number of patients who underwent special procedures (e.g., intravascular brachytherapy) was extracted from the Oncology Information System Mosaiq (Elekta Inc.; Crawley, UK) through database queries. We also included a task category called “other” to be able to include any effort not reflected in the previous categories, such as the average time spent on QA checks after repairs or upgrades. In cases where time estimates varied considerably among different physicists (i.e., tasks with a large range on their estimates), we reassessed the particular task through observation. The physicists with the minimum and maximum estimates performed the task together and arrived at a consensus. Any discrepancies in the estimates per task were discussed within the full physics group to ensure that average times were appropriate. From these estimates, we calculated the total time spent per month per task. We note this method of effort assessment essentially constitutes an independent validation of the workloads in that different physicists in the group agree as to the appropriate effort values assigned to each task. If each task is appropriately accounted for, then the overall workflow will also be reflective of actual effort.

### 2.2 Workload metric development

We introduced a metric we refer to here as the “equivalent workday” (eWD), to serve as a universal workload unit representing the ideal amount of work done by one person in 1 working day. For simplicity, we assumed 1 eWD equals 8 h. The eWD metric allows for conversion between the average time spent on each of the clinical tasks outlined above. Based on the above time estimates for each task, we determined the total effort (TE) for each physicist, p, who is responsible for tasks *t* = 1, 2,…, *n_p_*, using the following equation:

(1) $$\;TE_{p}=\sum_{t=1}^{n_{p}}({\mathrm{eWD})}_{t}\;/({\mathrm{cFTE})}_{p}$$

where (eWD)*_t_* is the 8‐h equivalent workdays necessary to accomplish each task, *t*, within a given month. TE*_p_* was computed for each physicist (*p* = 1, 2, ···, P). To assess the equity in the workload distribution, we computed the normalized TE (nTE) to a percentage, using the following equation:

(2) $$\;\mathrm{nT}E_{p}=\;\left(TE_{p}/\sum_{i\;=\;1}^{P}TE_{i}\right)\times 100\;\left(\%\right)$$

where $\sum_{i\;=\;1}^{P}TE_{i}$ is the total workload of the group consisting of *P* physicists.

### 2.3 Implementation of the effort system

The nTE metric enables quantifying and balancing the clinical workload between physicists—a central goal of this system. We can balance the nTE of each physicist by rearranging clinical duties and/or adjusting the number of days each person is assigned to common tasks such as Physicist‐Of‐the‐Day (POD), an assignment used in this clinic for primary physics clinical coverage on a given day. The latter is a particularly useful means of effort adjustment because POD is a task everyone performs and can be assigned to on a daily basis whereas the effort of larger assignments benefits from continuity, and therefore cannot be easily altered on a daily or monthly basis.

To better represent the total workload per physicist, we added two new tasks in our effort model to the list of tasks previously identified during the effort assessment. First, we added an eWD credit to account for vacation and professional leave (task “Leave”) to the nTE computation to ensure that physicists do not experience a “leave penalty” in a given month due to vacation or professional leave. We experimented with three different values for eWD per leave day—0.33, 0.4, and 0.8—during our trial period between October 2019 and June 2020 to find an appropriate empirical value. Note that the contribution of “Leave” credit in TE should not depend on the individual physicist's cFTE because the leave credit is applied to all responsibilities including clinical work, research, administrative work, and so forth. Therefore, Equation (1) is modified to

(3) $$\;TE_{p}={\left(\mathrm{eWD}\right)}_{\mathrm{Leave}}\;+\sum_{t\;=\;1}^{n_{p}}({\mathrm{eWD})}_{t}/({\mathrm{cFTE})}_{p}\mathrm{for}\;t\neq \mathrm{Leave}$$

where (eWD)_Leave_ comprises the number of eWDs of leave physicist *p* has for that given month. Another way to understand the first term in Equation (3) is that it represents the amount of clinical effort associated with each day of leave that a person takes, that is, (cFTE)*_p_* × (eWD)_Leave_. This total clinical leave time is then weighted by 1/(cFTE)*_p_* multiplicative factor like all other terms in Equation (1). This results in the first term in Equation (3).

We also added another category of tasks, “Clinical Project,” to account for various necessary clinical development or implementation projects undertaken by physicists. This type of task distinguishes itself from the other tasks as it is typically restricted to a time period of a few weeks or months. Examples of clinical projects are shown in Table 2. In this context, we also set up a process that allows physicists to apply for clinical project credits to proactively identify clinically relevant projects. Due to the differing nature of various clinical projects, we identified three distinct types of clinical projects and their corresponding pathways to allocate eWD credit. They are summarized in Table 1.

**TABLE 1:** Three different pathways to incorporate physics clinical project effort

Types	Description	eWD credits	Example project
Well‐defined project	The time required to complete a project can be estimated and the end goal is clear.	Physicist submits a request with estimated work hours, which are then converted to eWD credit.	Update of CT simulator QA program
Major equipment commissioning	The project requires a considerable time commitment to be completed and the need to complete the project is time sensitive. It applies to a specific period only, which needs to be defined before the project starts.	Physicist is exempt from clinical duties except the minimum clinical duties agreed upon by both the physicist and the Physics Effort Committee. Physicist monthly effort will not be balanced with others.	Linac or treatment planning system commissioning
Budget‐allocated project	The effort cannot be reasonably estimated before the project starts. Project is not time sensitive.	Physics Effort Committee assigns a budget eWD each month and the physicist works within the budget.	Introduction of a new patient physics consult program

eWD, equivalent workday; CT, computed tomography; QA, quality assurance.

### 2.4 Evaluation of the effort system

In order to evaluate the effort system, we assessed the impact on physics effort during two separate periods. The first period occurred between May 2018 and April 2019, during which the system described here was not used to assign clinical duties. Prior to this, tasks were assigned by one or a few people with no quantitative metric in place. The second period occurred between July 2020 and June 2021, wherein the system was fully operational. There was also a trial period between these two periods (between October 2019 and June 2020), during which certain parameters of the model were adjusted, most importantly the eWD values for leave credit and for the POD task. This was considered a trial period and is not included in the analysis reported here. In our clinic, the Physics Effort Committee is responsible for scheduling assignments and maintaining the database. The customized Microsoft Access database contains the eWD values and assignments of all tasks and leave days for each physicist in each month. Its read‐only version is available for all physicists to promote transparency. All data used for evaluating the effort system are from the Access database.

## 3 RESULTS

Table 2 shows an excerpt of the list of clinical tasks, including their description identified during the effort assessment. All physicists routinely perform the POD tasks, each of which was initially assigned a value of 1 eWD per day. Due to the complexity of operations, the POD was separated into two different categories—“POD1” and “POD2”—as shown in Table 2. In addition to POD, 10 other clinical tasks were identified, referred to here generically as Tasks 1–10. Note that we present an anonymized list of tasks since the specific eWD values for tasks in the clinic considered here will not translate to other clinics. Each clinic needs to determine appropriate eWD values for relevant tasks. Examples of tasks can be found in Table 2 for a subset of tasks to demonstrate the level of details included in the survey for accurate time estimates. Table 3 shows the calculations for task assignment and the data from the survey, which allows for a calculation of eWD per month for each task.

**TABLE 2:** Example of definition and scope of selected physics tasks to illustrate the level of details included in the survey for accurate time estimates

Task	Definition and scope
POD 1	Daily troubleshooting of four linacs Daily troubleshooting of two active breathing coordinator systems and treatment assistance as needed SBRT/mini TBI setup and treatment Patient‐specific in‐vivo dosimetry (IVD) measurement approval Emergent/unscheduled repairs/QA of four linacs between 6 AM and 4 PM End of treatment chart checks for an average of five patients Urgent plan checks for patients starting the next day before 10 AM Resident POD training
POD 2	New initial plan checks Emergent/unscheduled repairs/QA of four linacs between 4 PM and 10 PM Urgent IVD checks for patients having treatment before 9 AM the following day
Linac physicist	Monthly and annual QA for dosimetry and imaging system of one linac Linac meetings (monthly) Scheduled upgrades, repairs, and maintenance of a linac IVD system maintenance, calibration, upgrades, and testing Daily QA device maintenance, calibration, upgrades, and testing Linac‐specific part of commissioning/upgrades of TPS Mentoring one resident who is assigned to assist and learn
Low dose rate brachytherapy	Prostate seed implants—seed assay and inventory, treatment plan initial check, and treatment vault and patient survey before and after the procedure Eye plaques—seed assay and inventory, treatment planning, and post implantation QA

POD, physicist of the day; SBRT, stereotactic body radiation therapy; TBI, total body irradiation; IVD, in‐vivo dosimetry; QA, quality assurance; TPS, treatment planning system.

**TABLE 3:** Example of data from the survey to estimate the effort for each task

	Patient procedure		Monthly QA		Annual QA				
Tasks	No. of patients per year	Average length of procedure (h)	No. of QAs per year	Average time (h)	Average time (h)	Miscellaneous (h/month)	Total time spent (h/year)	eWD per year	eWD per month
Task 1	N/A	N/A	12	3	26.1	4.0	165.2	21	1.72
Task 2	N/A	N/A	12	4.5	1.0	1.0	73.7	9	0.77
Task 3	16	3	12	0	4.0	3.0	96.8	12	1.01
Task 4	90	2.75	0	0	1.0	3.0	284.5	36	2.96
Task 5	161	7	12	3	0.0	1.0	1175	147	12.24
Task 6	9	8	0	0	0.0	0.0	79.2	10	0.83
Task 7	N/A	N/A	12	1.5	0.0	2.0	42.0	5	0.44
Task 8	N/A	N/A	0	0	16.0	14.0	184.0	23	1.92
Task 9	N/A	N/A	12	10	0.0	1.0	132.0	17	1.38
Task 10	N/A	N/A	0	0	0.0	32.0	384.0	48	4.00

QA, quality assurance; eWD, equivalent workday.

**FIGURE 1:**
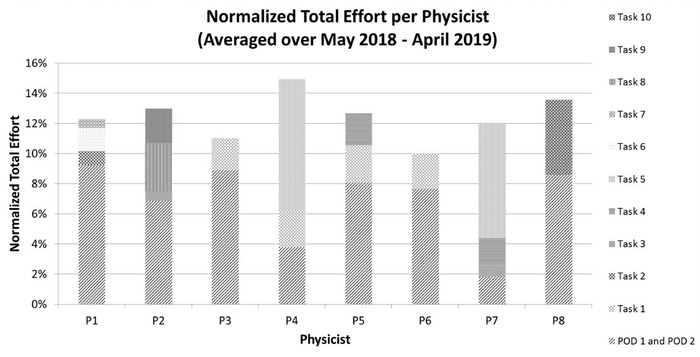
Normalized total effort for each physicist averaged over the 1‐year period of May 2018 to April 2019 before implementing the effort system

Figure 1 shows the relative distribution of the workload amongst physicists (nTE) for the 1‐year period of May 2018 to April 2019 (prior to when the system was developed). The nTE values for physicists ranged between 10.0% and 15.0%. For comparison, with an equitable workload distribution the expected nTE would be 12.5% for the group of eight physicists (*P* = 8). This indicates that physicist P4 with an nTE of 15.0% spent 50% more time on clinical duties compared to physicist P6 with an nTE of 10.0%.

During the trial period between October 2019 and June 2020, using a value of 0.8 eWD per leave day in the nTE computation was found to be optimal. This resulted in a total workload (i.e., the number of POD days plus other tasks), which was manageable during the months that included leave days. It was found that when a smaller eWD value for leave was used, physicists were assigned too many POD days during that month, leaving insufficient time to do the remaining tasks they were assigned, such as monthly Linac QA. A further adjustment that was implemented during this trial period was changes to the POD responsibilities. As a consequence, the eWD value per POD day was adjusted to 0.7.

**FIGURE 2:**
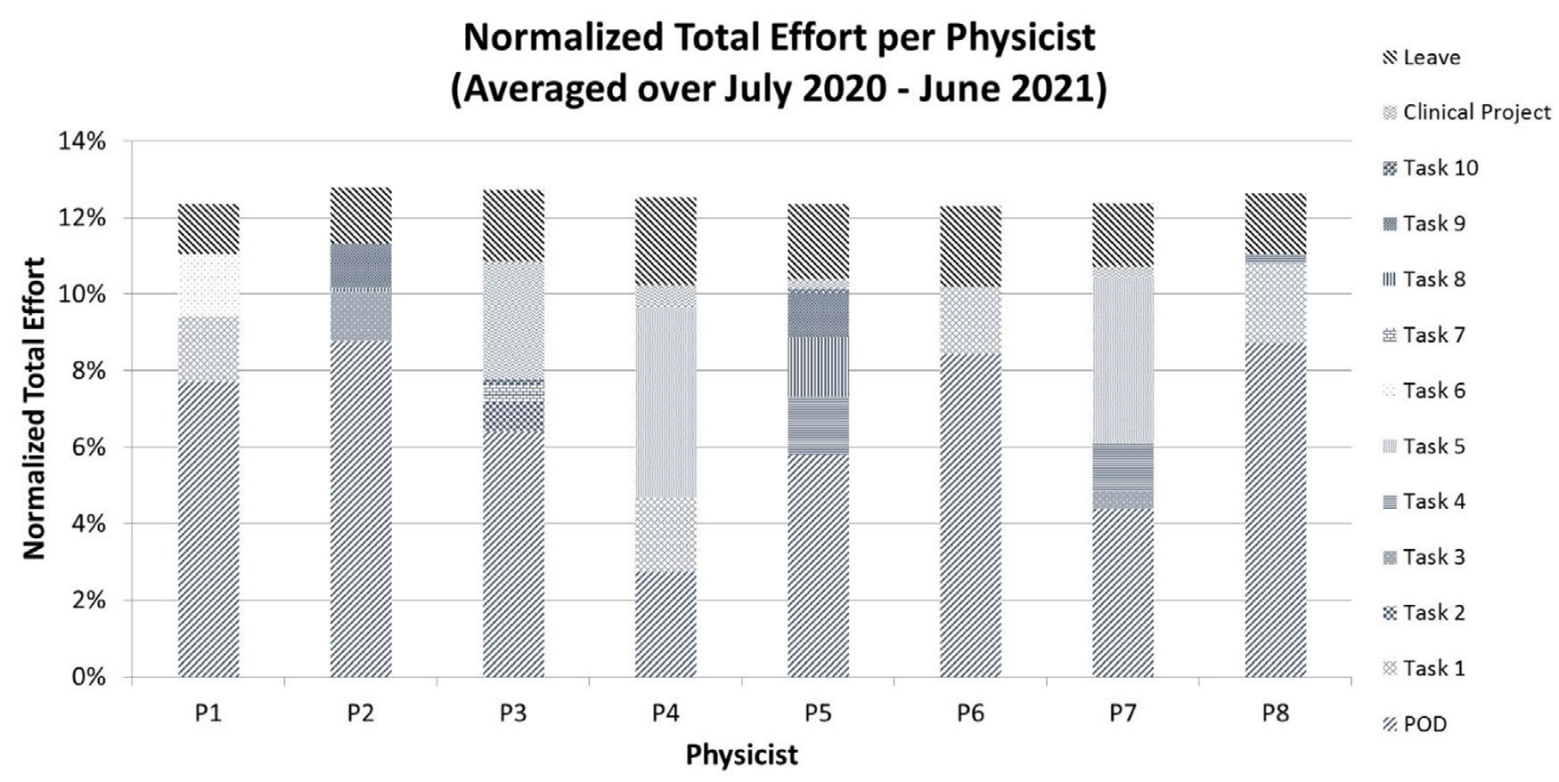
Normalized total effort for each physicist averaged over the 1‐year period of July 2020 to June 2021 after implementing the system

Figure 2 shows the average nTE distribution of all eight physicists for the 1‐year period after the system was fully deployed between July 2020 and June 2021. The nTE per physicist ranged between 12.3% and 12.8%.

## 4 DISCUSSIONS

We have developed a system to quantify the workload distribution for clinical medical physicists and demonstrated its feasibility in a radiation oncology clinic. The system utilizes a quantitative metric, nTE, which is based on an individual physicist's cFTE and a reference workload unit, eWD. This unit could be interpreted as a relative term to compare the workload among different physicists or as an absolute term to determine the total time spent on the clinical work by an individual physicist or the group. Implementing this system, we discovered that the previous task assignments that were performed without a quantitative metric were distributed unequally among physicists (10.0%–15.0% nTE), meaning that the clinical effort amongst physicists differed by up to 50% within the group. Though this was not intentional, it is perhaps not surprising given the complex, nonintuitive process of making assignments when the individual responsibilities within the group are not identical. After the implementation of this system, the effort was more equitably distributed with an nTE range of 12.3%–12.8%. It is important to note that the system provides for equity of overall workload but not the balance of workload between different tasks. For example, one goal might be to balance effort within the group such that each physicist contributes equally to clinical projects. However, the system does not enforce such a choice by construction. One can see this is not the case in Figure 2, where P4 had nearly half of the workload directed towards clinical projects while P2 had zero. This was a purposeful choice in our clinic based on the mutual interest of all physicists, but different choices are possible.

This system bears some resemblance to the RVUs currently used in the US healthcare system to reflect operative time, difficulty, skills, and stress for reimbursement. However, RVUs are limited to clinician providers. To our knowledge, no similar system has been described for medical physicists. Though RVUs are developed and tied to reimbursement, such a quantitative metric is helpful to determine the effort required to perform clinical tasks and equitable workload distribution in the medical physics group.

One advantage of the workload assessment described here is that it allows for inclusion of more complex ad‐hoc clinical projects that are needed to improve patient care; for example, updating our internal pacemaker policy in accordance with newly released AAPM task group recommendations^13^ or revamping procedures for the total body irradiation service. Such clinical projects were not listed as a task prior to the development of our system, and therefore no workload credit was explicitly conferred to the assignment although a considerable amount of time was needed to carry out those clinical projects. This can lead to a system with unrecognized efforts, which could make physicists reluctant to contribute to the clinical projects. By contrast, the effort system described here explicitly incorporates the effort needed for clinical projects, which provides transparency and additional incentive for physicists to undertake clinical projects.

There are a number of potentially attractive aspects of the effort system described here. First, it promotes transparency and accountability, and may help counteract implicit bias that results in gender or racial disparity in assignments and, potentially, promotions. Second, it allows for equitable workload balance in the case of illness, extended leave, or a situation where somebody leaves and the group is short of full‐time equivalents until a replacement is hired. In such situations, the workload can be spread out and shared by the entire group, avoiding extra burden for any one individual. Third, it is flexible and scalable in that it can include assignments in multiple sites of practice and distinct types of tasks, and accommodate different professional tracks (as long as the associated cFTEs are clearly defined). In situations where a physicist provides clinical service in multiple clinics, it may be useful to consider assigning a partial cFTE per clinic and have each clinic balance the workload within the clinic only rather than balancing the workload of all sites together. Fourth, our system accommodates each physicist's experience level in the assignment by allowing more than one physicist to perform the same task. When a physicist is assigned to a new task, which requires the supervision of a physicist with the required competencies, the total eWD values for all the physicists assigned is then included. Another variation would be a model with multiple task groups such that the workload can be equally distributed within each task group; for example, a nonroutine clinical project group and a routine clinical work group leading to a fair share of nonroutine and routine type of clinical tasks per physicist.

Implementing this effort system offered several lessons. First, there are many challenges in arriving at accurate time estimates for tasks. One example is sequential tasks. For example, if three stereotactic radiosurgery (SRS) patients are treated on a given day, they might not be treated back‐to‐back due to planned or unplanned delays. Therefore, multiplying the time it takes to treat one patient by three may not correctly reflect the time it takes to treat three patients. However, despite inherent uncertainty like this in the determination of the eWD value for each task, the system does ensure that everyone who performs the same task receives the same credit in their workload. To balance out any intrinsic inaccuracies in the eWD estimates, rotation of assigned tasks is an option. Second, for the tasks based on the number of patient procedures, such as low dose rate brachytherapy and SRS, the number of patients may vary over the timescale of months to years. Therefore, periodic updates of the patient numbers are necessary. An appropriate update interval also needs to be carefully chosen. We found that semi‐annually or annually is a reasonable frequency depending on the task.

Finally, the role of leadership is crucial in this undertaking. Leadership must be invested in the creation and maintenance of such an effort system. Leadership sets the expectation on which tasks are needed and prioritized in the clinic so they can be carried out using the available physics resources. This affects the overall workload of the entire group, not just those who performs the specific tasks, because the nTE is balanced for all members. Such careful discretion is particularly important in assigning clinical projects that require a long‐term commitment or compete with routine clinical work that cannot be delayed when physics staffing is limited.

There are several limitations to this study and issues to be aware of when attempting to apply it more broadly. The value of eWD for a given task depends strongly on the specific definition of the task, the tools available, and the workflow, all of which could vary widely among different institutions. It is prudent for each clinic to examine and determine a reasonable value of eWD for each task in their own clinic. Finally, this study was developed and evaluated in an academic therapy physics environment, so further validation is needed to assess whether the same principles apply to other areas of medical physics, such as diagnostic imaging or radiation safety.

## 5 CONCLUSION

We have developed and implemented a system for equitable workload distribution of clinical physics tasks. The system relies on a quantitative metric, eWD, which describes the effort required for each clinical task according to the specifics of operations in a given clinic, the support tools available, and clinical workflow and load. The system allows for various configurations to meet each clinic's own goal. It may be possible to use the total value of eWD to determine the need for physics staffing change when a new program or procedure is added to the department. The experience reported here from the evaluation period suggests that it is possible to quantify and equitably distribute the clinical workload. Moreover, a quantitative metric as that presented here allows for transparency and accountability. While this study has been developed and tested in an academic radiation oncology physics department, it may be generally applicable in other clinical environments by adjusting the cFTE or tasks as appropriate.

## AUTHOR CONTRIBUTIONS

Minsun Kim: As a Clinical Physics Lead at the University of Washington Medical Center, she participated in developing, refining, and implementing the system from the beginning to date. She drafted and revised the manuscript. Eric Ford: As a Vice Chair and Director of Medical Physics, he directed and implemented the developed system in the clinic. He provided valuable guidance in the direction of the manuscript and significantly contributed to the manuscript. Wade Smith: He joined the Physics Effort Committee after the development. He participated in refining and implementing the system. He provided feedback and edits to the manuscript. Stephen R. Bowen: He was a member of the initial Physics Effort Committee to develop the system and contributed to set it up. He provided feedback and edits to the manuscript. Sarah Geneser: She was a member of the initial Physics Effort Committee to develop the system and contributed to set it up. She provided feedback and edits to the manuscript. Juergen Meyer: He chaired the initial Physics Effort Committee to develop the system. He provided valuable guidance in the direction of the manuscript and significantly contributed to the manuscript.
